# The Effect of Mindfulness Interventions on the Sleep Quality of Pregnant Mothers in the Second and Third Trimesters of Pregnancy: Mindfulness and Sleep Quality in Pregnancy

**DOI:** 10.31661/gmj.v12i.2750

**Published:** 2023-12-17

**Authors:** Razieh Shamabadi, Mahboubeh Eslamzadeh, Elnaz Eskandarnia, Negar Asgharipour

**Affiliations:** ^1^ Psychiatry and Behavioral Sciences Research Center, Mashhad University of Medical Sciences, Mashhad, Iran; ^2^ Mashhad University of Medical Sciences, Mashhad, Iran

**Keywords:** Pregnancy, Mindfulness, Sleep Quality

## Abstract

**Background:**

Sleep disorder is a common problem during pregnancy and a large percentage of pregnant women (especially in the third trimester of pregnancy) experience changes in their natural sleep pattern. Considering the fact that sleep disorder can cause many complications before, during and after childbirth, it is necessary to achieve good sleep quality during pregnancy. Therefore, the present research investigated the effect of mindfulness interventions on the quality of sleep among pregnant mothers in the second and third trimesters of pregnancy.

**Materials and Methods:**

The present randomized clinical trial study used pre-education-post-education design in Sabzevar Health Center from 2019 to 2020 on all pregnant women referred for receiving prenatal care. A sample of 98 women who met the inclusion criteria were divided into intervention (n=50) and control groups (n=48). The sleep quality and depression anxiety and stress -21 (DASS-21) questionnaires, were completed by two groups at pre-test, post-test, and one month after the educational intervention. The intervention group received 6 sessions (1 hour and 30 minutes) of mindfulness lessons during 2 months, while the control group received routine pregnancy care and a mindfulness session was performed after completion of the post-education. Data was analyzed by SPSS software (version 20) and descriptive and repeated measures analysis were used.

**Results:**

Mindfulness interventions were effective in changing the sleep quality of pregnant mothers (P0.05). Also, the mean scores of anxiety, stress and depression questionnaires of pregnant mothers, as confounding variables on sleep quality, was significantly changed during different stages of the study (P0.05).

**Conclusion:**

Mindfulness training significantly increased the sleep quality of pregnant mothers. Therefore, mindfulness interventions can be used as effective interventions in improving psychological wellbeing during pregnancy and also reducing the anxiety and stress of pregnant women.

## Introduction

Sleep disorders are among the common problems during pregnancy affecting almost 80% of pregnant women worldwide [1]. The incidence of sleep disorders increases from the first to the third trimester of pregnancy [2][3]. Sleep problem complains in pregnancy may include difficulty falling asleep, frequent awakenings, reduced night sleep hours, reduced sleep efficiency, and sleepiness [4][5]. Decreased quality of sleep during pregnancy has harmful effects on individuals’ mood, cognitive performance, and general well-being [6]. A large percentage of pregnant women, especially in the third trimester of pregnancy, experience changes in the natural pattern of sleep. On the other hand, sleep disorders during pregnancy negatively affect other aspects of psychological wellbeing as the prevalence of depression, anxiety and stress is reported to be higher than general population during pregnancy [7].

Due to the fact that sleep disorders can cause various complications before, during and after childbirth, management of sleep disorders during pregnancy is an important issue [8]. Since one of the characteristics of a successful individual is to acquire the necessary skills for self-management in terms of behavior, emotion, and mind, the importance of managing the mind and thoughts becomes more apparent during pregnancy. Recent research showed a therapeutic role for mindfulness due to its positive and beneficial effect on many physical and mental problems, including chronic pain and stress disorders [9]. During mind-awareness, a person learns to be constantly aware of the state of his/her mind and to focus the attention on different ways of thinking [10]. Mindfulness practice, while changing the structure of the brain and making a person resistant to stressful factors, helps a person discover inner peace and vitality [11].

Cognitive therapy based on mindfulness is a new technique requiring metacognitive learning and new behavioral strategies to focus on attention, preventing rumination, and tendency to worrisome responses [12]. Since mental rumination during night sleep is strongly related to insomnia and depression during pregnancy and causes sleep disturbances, mindfulness interventions and relaxation exercises can potentially protect against these perinatal stress-related complications [13]. It has been demonstrated that mindfulness training can significantly reduce sleep deprivation in overweight mothers and were shown to improve depression, anxiety and stress in pregnancy [13][14][15][16]. However, the studies on the effectiveness of mindfulness trainings on the sleep quality and other psychological aspects of pregnant women are limited and their findings are inconclusive due to non-randomized designs and the use of different tools for assessing sleep quality in pregnancy [16]. The present study aimed to evaluate the effect of mindfulness training lessons on the sleep quality of pregnant women by conducting a randomized clinical trial.

## Materials and Methods

The present randomized clinical trial (RCT) was approved by the Mashhad Medical Sciences Ethic committee (Ethics ID: IR.MUMS.MEDICAL.REC.1398.572, IRCT number: IRCT20191204045601N1) and every pregnant woman who referred to Sabzevar Health Center between 2019 and 2020 to receive prenatal care were evaluated. The inclusion criteria were as follows:

• Being able to participate in educational programs based on the psychologist opinion

• Not applying for similar educational classes

• Absence of chronic medical diseases (diabetes, blood pressure, kidney diseases, asthma, etc.)

• Not having a history of any psychiatric disorders

• Having at least a diploma degree since the education intervention required secondary education

• Having a minimum age of 20 and a maximum age of 39 years

• Having poor sleep quality (Pittsburgh sleep quality score higher than 5)

The exclusion criteria were as follows:

• Not willing to continue the study

• Becoming a high-risk pregnancy (risk of miscarriage and bleeding)

• Premature birth (birth earlier than 38 weeks)

• Experiencing acute stressful events during the study period

• Failure to regularly attend training sessions and failure to practice at home.

According to the previous study by Farahbakhsh et al. [17] and the following assumptions, the sample size in the intervention group was 50 people and the control group was 48 people (drop of rate of 8% for each group was considered).


\left[ Z_{(1-\alpha/2)} + Z_{(1-\beta)} \right]^2 \quad \alpha = 0.05 \quad
                    Z_{(1-\alpha/2)} = 1.96 \quad \beta = 0.21

The included pregnant women were randomly allocation to intervention (n=50) and control (n=48) groups. For both groups, pre- and post-intervention questionnaires were completed at the beginning and end of the mindfulness interventions. Routine pregnancy care was also performed for the intervention and control groups.

Training sessions for the intervention group consisted of 6 sessions (1 hour and 30 minutes) during 2 months at specified times by a specialized assistant in psychiatry and under the supervision of relevant educational specialist. The control group only received one mindfulness lesson.

The sessions were presented in a virtual form using the Telegram application (www.telegram.org). Moreover, a training compact disk (CD) was provided to the intervention group participants for each session. It is worth mentioning that due to the special conditions of Corona virus outbreak and the vulnerability of pregnant mothers to this disease, the meetings for both the intervention and control groups were held in absentia and through the virtual space. After the sessions, post-intervention tests were done for both groups. The training sessions were presented as follows:

First session: Auto-guidance (When we notice auto-guidance, we find the presence of mind). This session aimed at becoming aware of the auto-guidance as the autopilot in self-control. A great way to take back control is to take time out for a mindfulness meditation and to practice a simple eight-minute practice designed to focus on the present time.

Second session: Focusing more on the body (focusing on the body makes the mental whispers more obvious and leads to more control of the reaction to daily events). This session was designed to focus on one’s body. However, states and emotions are a little denser and it is very easy to ignore them. The purpose of the fourteen-minute "body scan" meditation was to open the communication channels between body and mind. Follow the same meditation routine as before, imagining that every part of the body that is focused on is being inflated with each inhale and empty with each exhale. Paying attention to one’s feeling while tingling one’s feet as an example. Remembering that there is no winning or losing or success or failure. If participants are distracted, they were instructed to regain concentrate by performing exercise and practice the exercise twice a week. Third session: Knowing how the mind can often be busy and distracted, we learn to deliberately focus our awareness on breathing to provide the possibility of being more focused and integrated. In the third session, "three-minute breathing" meditation was instructed, which should be performed twice a day. In this step, participants took two minutes to focus on their feelings, thoughts and body, then one minute of deep breathing and focusing on inhaling and exhaling.

Fourth session: Most of the time, when the mind wants to focus on one subject and avoid other subjects, it gets distracted. In the presence of mind approach to be present simultaneously, a person must look at events from a different angle in order to obtain a broad and different view of these events. In this session, participants learned how to distance themselves from their thoughts by practicing "Sounds and Thoughts" exercise. In this exercise took participants try to do nothing for eight minutes and only pay attention to the sounds around them. If the exercise is performed correctly, one finds that the sounds come and go like the tides of thoughts. In the state of complete focus, mind starts to tell stories based on the sounds; For example, from a loud sound, one gets the impression that a cement brick has fallen from the roof. This meditation is a great way to learn how mind works and makes participants feel more relaxed with the flow and nature of their thoughts.

Fifth session: Different communication means allowing oneself to be present to the experience, exactly as it is, without judging it or trying to make a change. The exercise that was thought in this session was called "searching for difficulties". Participants were instructed to perform the exercise for ten minutes every day. To do the exercise participants were instructed to get comfortable and whenever they were ready, they were asked to think about a difficult or unpleasant subject. Then, they should try to find out where they feel the thoughts in their bodies, as soon as they found that spot, while taking a deep breath, leting those feelings absorb. This is the moment of acceptance and compassion that prepares participants to be free and comfortable. Participants were asked to combine this meditation with the meditations they have learned before: "Breath and Body", "Sounds and Thoughts", "Looking for Difficulties" and "Three Minute Breathing".

Sixth session: Participants were instructed about how negative moods and thoughts limit one’s connection with experience. It is reasonable to understand that thoughts are just thoughts, even for someone who does not believe in this. This session dealt with memory. A memory is a tendency to remember events from the past, in a completely negative way. For example, one may feel like his/her entire high school experience was awful due to having one awkward class experience. When one feels and behaves like this, one easily blames oneself and others. But blaming does not help reconcile with the past. In fact, research has shown that it makes it even more difficult.

### 1. Study Questionnaires

In this study, the following questionnaires were used to measure clinical and demographic characteristics:

### 1.1.Pittsburgh Sleep Quality Questionnaire:

This questionnaire measures a person's sleep habits during the past month in seven domains, including mental quality of sleep, delay in falling asleep, sleep duration, efficacy of sleep, sleep disorder, use of sleeping pills, and daily dysfunction. The questionnaire items are scored in a Likert scale of zero, indicating none, one, indicating less than once a week, two, indicating twice a week, and three indicating more than three times a week. The total score can range between 0 and 39, the higher the sleep quality score, the lower the sleep quality. The Pittsburgh Sleep Quality Questionnaire (PSQI) was designed and validated by Boyce and colleagues, who reported that the internal consistency of the questionnaire using Cronbach's alpha was 0.83. The Persian version of this questionnaire was validated on 125 patients in a study by Farrahi Moghadam et al. and the Cronbach's alpha coefficient was reported to be 0.77 for all subjects [18].

### 1.2. Questionnaire DASS-21 (Depression, Anxiety, Stress)

Depression, anxiety and stress questionnaire-21 (DASS-21) consists of 21 statements related to the symptoms of negative emotions (depression, anxiety and stress). The depression subscale includes expressions that measure unhappy mood, lack of self-confidence, hopelessness, worthlessness of life, lack of interest in involvement in affairs, lack of enjoyment from life, and lack of energy and strength. The anxiety subscale evaluates physiological overexcitement, fears and situational anxiety, and the stress subscale evaluates expressions such as difficulty in achieving relaxation, nervous tension, irritability and restlessness. In this questionnaire, participant should rate the frequency of the mentioned symptoms during the last week using a 4-point Likert scale (ranging between 0 and 3). Each of the three scales of depression, anxiety and stress has 7 questions. The total Cronbach's alpha score for DASS-21 was 0.70 and the internal consistency of the Persian version of DASS-21 was 0.77 for depression, 0.79 for anxiety and 0.78 for stress [19].

### 2. Ethical Consideration:

The present study was registered in the Iranian Clinical Trial Registration Center (IRCT20191204045601N1). In order to comply with ethical considerations, before conducting the interview process and completing the checklist, the level of health and acceptability of the patient's general judgment were considered. The written consent was obtained from the participants or their guardian prior to entering the study.

### 3. Statistical Analysis

Data analysis was done using the statistical package for social sciences (SPSS) version 20 (SPSS Inc., Chicago, Ill., USA). Continuous variables were presented using mean and standard deviation. Comparison of the mean variables between groups was done using independent t-test for continuous variables and chi square for categorical variables. Comparison of the mean values of the study parameters between study time points was done using the paired t-test. The univariate repeated measures analysis of variance using Wilks Lambda was performed to evaluate the effect of time, group, and time-group interaction after adjusting for confounders. The level of statistical significance was 0.05.

## Results

**Table:** Table1. Demographic Characteristics of the Participants and Their comparison between study groups

Variable		Intervention	Control	p
Age (years)		28.09 ± 5.44	28.08 ± 5.89	0.105
Gestational age	2^nd^ trimester	28 (58.2%)	28 (58.2%)	0.21
	3^rd^ trimester	29 (58.1%)	20 (41.8%)	
Level of education	Highschool graduate	19 (38%)	17 (35.4%)	0.072
	Diploma	6 (12%)	3 (6.2%)	
	Masters	25 (50%)	28 (58.4%)	

**Table:** Table2. Mean Depression, Stress, Anxiety, and Sleep Scores at Baseline and at the End of the Study in the Study Groups

Variable	Group	Pre-test	Post-test	p	Conclusion
Depression	intervention	5.56 ± 2.9	4.66 ± 4.1	0.126	The mean depression score did not significantly change after the intervention.
	Control	7.89 ± 3.73	8.2 ± 4.51	0.12	Increased depression score was not statistically significant.
Stress	intervention	9.27 ± 3.45	3.46 ± 2.84	<0.001*	Mean stress score significantly decreased after intervention.
	Control	11.76 ± 4.88	6.58 ± 4.65	<0.001*	Mean stress score was significantly reduced.
Anxiety	intervention	7.77 ± 2.61	3.04 ± 2.88	<0.001*	Mean anxiety score significantly decreased after intervention.
	Control	9.7 ± 3.56	5.54 ± 3.6	<0.001*	Mean anxiety score was significantly reduced.
Sleep	intervention	7.40 ±2.11	2.52 ± 1.59	<0.001*	Mean sleep score significantly decreased after intervention.
	Control	8.12 ± 1.85	6.4 ± 3.46	<0.001*	Mean sleep score was significantly reduced.

**Table:** Table3. Univariate Repeated Measures Analysis of Variance for Time and Group Interactions for Sleep Quality

Between group effect	Test	Value	F	df (Error)	df (Effect)	p	Eta square
Intervention	Wilk's lambda	0.892	11.4	94	1	0.001*	0.108
Control	Wilk's lambda	0.506	91.718	94	1	0.001*	0.494

**Table:** Table4. Between and Within Group Differences in Sleep Quality based on Univariate Repeated Measures Analysis of Variance

Reference	Sum of squares	df	Mean square	F	p	Eta square
Between group						
Group	579.378	1	579.378	91.718	<0.001*	0.494
Error	1088.372	100	10.884			
Within group						
Time	72.012	1	72.012	11.4	<0.001*	0.108
Time * group	579.378	1	579.378	91.718	<0.001*	0.494
Error (time)	593.794	94	6.317			

**Table:** Table5. Univariate Repeated Measures Analysis of Variance for Time and Group Interactions for Stress Score

Between group effect	Test	Value	F	df (Error)	df (Effect)	p	Eta square
Intervention	Wilk's lambda	0.487	105.258	100	1	<0.001*	0.513
Control	Wilk's lambda	1.000	0.017	100	1	0.898	<0.001

**Table:** Table6. Between and Within Group Differences in Stress Score based on Univariate Repeated Measures Analysis of Variance

Reference	Sum of squares	df	Mean square	F	p	Eta square
Between group						
Group	0.147	1	0.147	0.007	0.931	<0.001
Error	1957.591	100	19.576			
Within group						
Time	1547.456	1	1547.258	105.258	<0.001*	0.513
Time * group	0.245	1	0.245	0.017	0.898	<0.001
Error (time)	1470.161	100	14.702			

**Table:** Table7. Univariate Repeated Measures Analysis of Variance for Time and Group Interactions for Anxiety Score

Between group effect	Test	Value	F	df (Error)	df (Effect)	p	Eta square
Intervention	Wilk's lambda	0.496	101.544	100	1	<0.001*	0.504
Control	Wilk's lambda	0.985	1.548	100	1	0.216	0.015

**Table:** Table8. Between and Within Group Differences in Stress Score based on Univariate Repeated Measures Analysis of Variance

Reference	Sum of squares	df	Mean square	F	p	Eta square
Between groups						
Group	1.174	1	1.174	0.103	0.749	0.001
Error	1140.082	100	11.401			
Within group						
Time	959.462	1	959.462	101.544	<0.001*	0.504
Time * group	14.626	1	14.626	1.548	0.216	0.015
Error (time)	944.872	100	9.449			

**Table:** Table9. Univariate Repeated Measures Analysis of Variance for Time and Group Interactions for Depression Score

Between group effect	Test	Value	F	df (Error)	df (Effect)	p	Eta square
Intervention	Wilk's lambda	0.468	113.78	100	1	<0.001*	0.532
Control	Wilk's lambda	0.997	0.356	100	1	0.005*	0.447

A total of 98 participants (50 in the intervention and 48 in the control groups) were included in the study. Demographic characteristics of the participants are presented and compared between study groups in Table-1. There was no significant difference in age (P=0.105), gestational age (P=0.210), level of education (P=0.072) between the intervention and control groups. The mean age of the participants in the intervention and control groups were 28.09 ± 5.44 and 28.08 ± 5.89 years, respectively. There was no significant difference between the groups in terms of age (P=0.105) and level of education (P=0.072). Among the participants in the intervention group, 28 (58.2%) were in the second and 29 (58%) were in the third pregnancy trimester, while 28 (58.2%) participants were in the second and 20 (41.8%) were in the third pregnancy trimester in the control group. There was no significant difference between groups in terms of mean gestational age (P=0.21). The mean scores for study variables at pre-test and post-test are shown in Table-2. There was a significant difference in mean stress, anxiety, and sleep scores between pre-test and post-test measurements. Mean depression score did not change significantly in study groups.

### Effects of Mindfulness on Sleep Quality of Pregnant Women

The Wilks Lambda results showed that sleep quality was significantly different between pre-test and post-test. This finding indicated a significant interaction between time and group in terms of sleep quality (P<0.05, Table-3). Between group and within group comparison of sleep quality was evaluated using the univariate repeated measures ANOVA (Table-4). The effect size (eta square) for time and time-group interaction were 0.108 and 0.494, respectively (P<0.05). The mindfulness intervention was effective in changing sleep quality. In other words, the mean sleep quality scores significantly changed between time points in this study. This indicates that improvement in mindfulness skills of the participants (intervention and control groups) affected sleep quality over time. There was a significant difference between groups at pre-test (P=0.003).

### Effects of Mindfulness on the Stress of Pregnant Women

The results of univariate repeated measures ANOVA for stress are shown in Table-5. The Wilks Lambda results showed that stress score was significantly different between pre-test and post-test (P<0.05). On the other hand, there was no significant interaction between time (pre-test and post-test) and group (intervention and control) (P>0.05). Between group and within group comparison of stress score was evaluated using the univariate repeated measures ANOVA (Table-6). The mindfulness intervention was effective in changing the stress scores. The effect size (eta square) for time and time-group interaction were 0.513 and <0.001. Therefore, a significant change was seen in stress scores over the study time points. This finding indicated that improvement in mindfulness skills in the participants (intervention and control groups) significantly affected stress scores over time.

### Effects of Mindfulness on the Anxiety of Pregnant Women

The results of univariate repeated measures ANOVA for anxiety are shown in Table-7. There was a significant difference in anxiety scores between times (pre-test and post-test). On the other hand, there was no significant difference in anxiety scores between times (pre-test and post-test) and groups (intervention and control groups) (P>0.05). Between group and within group comparison of anxiety score was evaluated using the univariate repeated measures ANOVA (Table-8). There was a significant difference in anxiety scores between pre-test and post-test, while no significant difference was seen between intervention and control groups (P>0.05). This finding indicated that the mindfulness intervention was effective on anxiety scores. The effect size (eta square) for time and time-group interaction were 0.504 and 0.015, respectively. Therefore, anxiety scores significantly changed in both the intervention and control groups over time.

### Effects of Mindfulness on the Depression of Pregnant Women

The results of univariate repeated measures ANOVA for depression are shown in Table-9. The mean depression score was significantly higher in the control group compared to the intervention group both at pre-test and post-test measurements. Between group and within group comparison of anxiety score was evaluated using the univariate repeated measures ANOVA (Table-9). There was a significant difference in depression scores between times (pre-test and post-test) and groups (intervention and control groups) (P<0.05). The effect size (eta square) for time and time-group interaction were 0.532 and 0.497, respectively. Therefore, the mean depression score significantly changed in both the intervention and control groups over time. This finding showed that improvement in mindfulness skills affected depression scores of the participants in both groups (intervention and control groups) over time.

## Discussion

The present study demonstrated that mindfulness interventions were effective in changing the sleep quality of pregnant mothers. Moreover, mindfulness interventions significantly improved the average score of anxiety, stress and depression, as a confounding variable on the quality of sleep, among pregnant mothers in different stages of the study.

The findings of previous studies in terms of the effectiveness of mindfulness on sleep quality in pregnancy has been controversial. A study by Kantrowitz-Gordon et al. evaluated the feasibility, acceptability and initial effectiveness of online mindfulness intervention on the sleep of 50 pregnant women with gestational age of 12 to 28 weeks suffering from insomnia. Sleep quality was measured using the e-Pittsburgh Sleep Quality Index questionnaire, and the total sleep duration, wakefulness duration, fatigue, stress, anxiety, depression, and the positive impact on life were also evaluated in the study. Similar to our study, they reported that mindfulness intervention was effective in improving sleep quality in pregnancy [20]. A similar study evaluated the effects of mindfulness intervention on global sleep quality, depressive symptoms and perceived stress among overweight and low-income pregnant women and showed that mindfulness interventions significantly improved the sleep quality of women and reduced their stress symptoms caused by poor sleep quality at the end of the study. However, depressive symptoms caused by poor sleep quality was not affected by the intervention [14].

However, unlike our study, this study did not find a significant difference in the sleep quality of pregnant mothers in both case and control groups. The reason for this difference might be related to the racial diversity and differences in the socioeconomic status of the participants which could have reduced the effect of mindfulness intervention [14]. Similar to the findings of the present study, Lee et al. evaluated the effects of weekly mindfulness and hatha yoga classes on 15 healthy women in the second and third trimesters of their first pregnancy and demonstrated that women receiving the intervention in their second trimester had less night awakenings and sleep disorders than women who started in the third trimester [21]. Although the mentioned study evaluated sleep quality using the sleep disturbance scale (GSDS) and used yoga intervention besides mindfulness, its findings were similar to the findings of the present study.

Previous studies indicated that the time of initiating interventions might also affect sleep quality. For instance, a study on 19 healthy pregnant women showed a clinically significant improvement in sleep and pain in women who started yoga in their second trimester, while performing the intervention in the third trimester only improved stress and anxiety [22]. In the present study pregnant women in their second and third trimester were included and received the intervention in a similar manner. However, the intervention was effective in both groups. The reason for this difference might be attributed to the higher sample size in the present study compared to the mentioned study and also to the differences in conducting the intervention between the studies.

Mindfulness intervention was implemented in different ways. For instance, mindfulness intervention using M-Yoga (mindfulness yoga) was effective in reducing the symptoms of depression and scored of mindfulness and maternofetal attachment in a study on 18 women in their 12th to 26th week of gestation [23]. These findings were in line with the findings of the present study in terms of the effects of mindfulness on depression; however, the mentioned study did not evaluate sleep quality and other mental states in pregnant women [23].

Similar to previous studies that demonstrated the effects of mindfulness on the confouncders of sleep quality, namely stress, anxiety and depression, among pregnant women, the results of the present study indicated an improvement in the mean scores of these mental conditions in pregnant women who received mindfulness intervention [24][25]. Considering the different tools used for assessing mental state, the positive effect of mindfulness in previous studies as well as the present study might indicate the strong effect of this intervention on mental state of pregnant women.

## Conclusion

The present study demonstrated that mindfulness interventions significantly changed the quality of sleep in pregnant women and therefore, it can be concluded that using mindfulness skills during their sleep time improve psychological wellbeing.

## Conflict of Interest

None to declare.
